# Effects of combined epidural and general anesthesia on intraoperative hemodynamic responses, postoperative cellular immunity, and prognosis in patients with gallbladder cancer: A randomized controlled trial

**DOI:** 10.1097/MD.0000000000006137

**Published:** 2017-03-10

**Authors:** Jun Zhu, Xue-Rong Zhang, Hu Yang

**Affiliations:** Department of Anesthesiology, The Xinjiang Uygur Autonomous Region People's Hospital, Urumqi, P.R. China.

**Keywords:** cellular immunity, combined epidural-general anesthesia, gallbladder cancer, general anesthesia, hemodynamic responses, prognosis

## Abstract

**Background:**

This study is supposed to investigate the effects of combined epidural and general anesthesia on intraoperative hemodynamic responses, postoperative cellular immunity, and prognosis in patients with gallbladder cancer (GBC).

**Methods:**

One hundred forty-four GBC patients were selected and randomly divided into the general anesthesia (GA) group and the combined epidural-general anesthesia (CEGA) group. Before anesthesia induction (t0), at intubation (t1), at the beginning of surgery (t2), 5 minutes after pneumoperitoneum (t3), at the end of surgery (t4), after recovery of spontaneous breathing (t5), after regaining consciousness (t6), and after extubation (t7), the heart rate (HR), systolic blood pressure (SBP), diastolic blood pressure (DBP), and the depth of anesthesia (bispectral index [BIS]) were detected. Blood samples were separately collected 30 minutes before anesthesia induction (T1), 2 hours after the beginning of surgery (T2), at the end of surgery (T3), 1 day after surgery (T4), 3 days after surgery (T5). The survival rates of T cell subsets (CD3+, CD4+, CD8+, CD4+/CD8+) and natural killer (NK) cells were determined by flow cytometry. Postoperative nausea and vomiting (PONV), visual analog scale (VAS), and sedation-agitation scale (SAS) were performed to assess postoperative adverse reactions. A 3-year follow-up was conducted.

**Results:**

Compared with the GA group, the CEGA group had significant lower SBP values at t5 and t6, lower DBP values at t1, t3, t4, and t5, lower HR values at t1 and t5, and higher BIS values at t4, t5, t6, and t7. No PONV was observed in the CEGA group. In comparison to the GA group, the VAS was markedly increased and survival rates of CD3+, CD4+, and CD4+/CD8+ cells were increased at T2, T3, T4, and T5 in the CEGA group. The 1-year, 2-year, and 3-year survival rates were not evidently different between the CEGA group and the GA group.

**Conclusion:**

Our study provides evidence that the combined epidural-general anesthesia might attenuate intraoperative hemodynamic responses and improve postoperative cellular immunity, so that it might be a more available anesthesia method for GBC patients.

## 1 Introduction

Gallbladder cancer (GBC), a common malignant tumor in digestive system, has a high mortality rate and lacks diagnostic specificity, which leads to a great challenge of preoperative and early-stage diagnosis.^[1]^ Although GBC is identified as a relatively rare tumor, it shows extensively poor prognosis.^[2]^ Currently, surgical resection remains the mainstay for the treatments of GBC patients, such as laparoscopic cholecystectomy, radical mastectomy, and extended radical mastectomy, and the latter 2 methods are more effective to improve prognosis.^[3,4]^ The survival rate was reported to be closely related to the cellular immunity. The inhibition of cellular immunity was mainly achieved through the interaction of nervous system, endocrine system, and immune system.^[5]^ The immune cells secreted and released a variety of neuropeptide hormones, endocrine hormones, and receptors (endogenous opioid, nonopioid peptide, cortisol, and catecholamine).^[6,7]^ Surgical anesthesia methods might be closely related to cellular immunity in the body, and all these hormones and receptors mentioned above could inhibit the activities of immune cells, natural killer (NK) cells, and humoral immune cells.^[8,9]^

The anesthesia method for surgery has been a major concern in plenty of cancer researches.^[10–12]^ General anesthesia (GA), local anesthesia, and other anesthesia methods had significant effects on postoperative immune cells activity, which may lead to tumor recurrence and metastasis so as to affect prognosis through suppressing immune cell activity.^[13]^ Zhao et al^[14]^ pointed out that either general anesthesia or epidural anesthesia influences cellular immunity during surgical stimulation. Epidural anesthesia is one of common methods for intraoperative and postoperative analgesia. However, it may easily lead to repetitive changes in blood pressure and heart rate (HR) during surgery, which induces hypotension or abnormalities of heart rate.^[15]^ The hemodynamic assessment has been applied to monitor the blood pressure during the period of anesthesia, and the stability of hemodynamic responses is considered an indicator of surgical outcome.^[16,17]^ The present clinical surgeries for GBC patients were generally applied with either general anesthesia or epidural anesthesia. But limited reports concerned about the effect of combined epidural-general anesthesia on the blood pressure, cellular immune activity for GBC patients. This study aims to investigate the effects of combined epidural and general anesthesia on intraoperative hemodynamic responses, postoperative cellular immunity, and prognosis in patients with GBC, so as to provide a promising improvement of postoperative prognosis.

## 2 Materials and methods

### 2.1 Ethical statement

This clinical study was performed with the approval of the Ethics Committee at the Xinjiang Uygur Autonomous Region People's Hospital. Informed consents were collected from all patients in this research.

### 2.2 Study subjects

A total of 144 patients with GBC were selected in the Xinjiang Uygur Autonomous Region People's Hospital from January 2010 to January 2012. Inclusion criteria: patients were younger than 80 and were classified as American Society of Anesthesiologists (ASA) I or II according to the ASA classification.^[18]^ Patients were pathologically confirmed as GBC during or after the surgery. Patients received laparoscopic cholecystectomy. Patients were diagnosed as stage I to IV GBC according to the tumor-node-metastasis (TNM) staging of the American Joint Committee on Cancer (AJCC).^[19]^ Patients had no history of endocrine disease, and had not received any radiotherapy, chemotherapy, hormonotherapy, or blood transfusion before surgery. Exclusion criteria were hematopoietic dysfunction, autoimmune disease, immunodeficiency diseases, severe coronary heart disease, or (and) hypertension. Besides, emergency patients or patients unwilling to participate were excluded. There were 86 female patients and 58 male patients, aged 31 to 76 years, with a mean age of (47.85 ± 6.61) years. All included patients were divided into the combined epidural and general anesthesia (CEGA) group (n = 72) and the GA group (n = 72). There were 30 male patients and 42 female patients in the GA group, whose mean age was (48.15 ± 6.89) years. There were 28 males and 44 females in the CEGA group, whose mean age was (47.01 ± 6.31) years.

### 2.3 Surgical approaches

Among all the 144 patients, patients with stage I GBC underwent laparoscopic cholecystectomy. And patients with stage II to IV GBC underwent laparoscopic radical cholecystectomy, followed by the resection of hepatic tissue within 2 cm around the gallbladder bed, and lymph node dissection that includes removal of the lymph nodes in the gallbladder, around the hilar, around the common bile duct, retroperitoneum, in the hepatoduodenal ligament, around the common hepatic artery, and at the posterior superior region of the pancreas head.

### 2.4 Anesthesia methods

Before anesthesia, all patients were injected with sodium lactate ringer solution. After intravenous infusion of Atropine (0.5 mg), pure oxygen inhalation was conducted via a facial mask at 6 L/min. The epidural puncture at the site of interspinous space between thoracic vertebrae 10 and 11 was performed for patients in the CEGA group, where 5 mL of 0.5% levobupivacaine was injected (50 mg/10 mL, Zhuhai Rundu Mintong Pharmaceutical Co, Ltd, Guangdong, China) with a tube inserted 4 cm above the puncture point. After the epidural block was identified as successful, an additional dose of local anesthetics (5–10 mL) was injected into patients via a puncture tube. When the level of anesthesia was up to the expected level for the surgery, the induction of general anesthesia was conducted. The agents for the general anesthesia included 0.1 mg/kg of vecuronium bromide (Zhejiang Xianju Pharmaceutical Co, Ltd, Zhejiang, China), 1 ug/kg of remifentanil (Hubei Yichang Human-well Pharmaceutical Co, Ltd, Hubei, China), and 0.2 mg/kg of midazolam (Jiangsu Nhwa Pharmaceutical Co, Ltd, Xuzhou, Jiangsu, China). The mechanical ventilation was performed after the intubation. Patients received an intravenous injection of vecuronium bromide 0.1 ug/(kg·min) and remifentanil 0.1 ug/(kg·min) to maintain anesthesia, followed by continuous inhalation of 1%–3% sevoflurane (Maruishi Pharmaceutical Co, Ltd, Osaka, Japan). The injection of muscle relaxants was terminated 30 minutes before the end of surgery, and the injection of all anesthetics was terminated 10 minutes before the end of surgery. The induction and maintenance of anesthesia for patients in the GA group were performed as same as the CEGA group, except epidural block. Patients of both 2 groups received routine intravenous infusion.

### 2.5 Hemodynamic assessment

During anesthesia, the multifunctional anesthesia monitoring equipment was used to detect heart rate (HR), systolic blood pressure (SBP), diastolic blood pressure (DBP), and the depth of anesthesia (bispectral index [BIS]). The values of SBP, DBP, HR, and BIS were observed and recorded at 7 different time points: before anesthesia induction (t0), at intubation (t1), at the beginning of surgery (t2), 5 minutes after pneumoperitoneum (t3), at the end of surgery (t4), after recovery of spontaneous breathing (t5), after regaining consciousness (t6), and after extubation (t7). All patients received treatment of blood pressure control before the surgery. And timely rescue measures would be taken when adverse reactions occurred during the surgery.

### 2.6 Flow cytometry

Vein blood sample (6 mL each patient) was extracted with intravenous catheter respectively at the following 5 time points: 30 minutes before anesthesia induction (T1), 2 hours after the beginning of surgery (T2), at the end of surgery (T3), 1 day after surgery (T4), 3 days after surgery (T5). Each vein blood sample was dispensed into 2 tubes. The 4 mL sample was coagulated in a silicone tube, followed by a 15-minute centrifugation (2000 r/min) at 4°C with the Beckman Coulter high-speed centrifuge (Beckman Coulter Inc, Hialeah, FL). Then the serum was extracted, and placed into an Eppendorf (EP) tube, labeled and preserved in a −70°C freezer (Sanyo, −80°C cryogenic refrigerator). Meanwhile, the other 2 mL blood sample was loaded into an Ethylene Diamine Tetraacetic Acid (EDTA) anticoagulant tube and stored for no more than 24 hours at 4°C. A 3-color flow cytometer (Beckman Coulter FC500; Beckman Coulter Inc, Hialeah, FL) was used to analyze the survival rates of T cell subsets (CD3+, CD4+, CD8+, CD4+/CD8+) and natural killer (NK) cells (CD3+/CD16+CD56+). The numbers of T cell subsets were measured using PerCP-labeled mouse antihuman CD3, Fluorescein isothiocyanate (FITC)-labeled mouse anti-human CD4, and PE-labeled mouse antihuman CD8 monoclonal antibodies (Becton, Dickinson and Company, Franklin Lakes, NJ). And the numbers of NK cells were detected with the FITC-labeled mouse antihuman CD3 and PE-labeled mouse antihuman CD16/56 monoclonal antibodies (Beckman Coulter Inc, Hialeah, FL). CD4 (%) = the number of (CD3+ CD4+)/the number of CD3 total T cells. CD8 (%) = the number of (CD3+ CD8+)/the number of CD3 total T cells. NK cells (%) = the number of NK cells in the right upper quadrant/(the number of NK cells in the right upper quadrant + the number of NK cells in the right lower quadrant) × 100%.

### 2.7 Postoperative adverse reactions

The incidence rates of postoperative nausea and vomiting (PONV), bucking, dizziness, and chest tightness were measured 12 hours after the surgery. Tropisetron was used for symptomatic treatment when vomiting occurred, and the dosage was recorded.

Visual analog scale (VAS) was applied to assess the degree of pain and dizziness of patients after 12 hours of the surgery. The assignments were as follows: 0 as no pain, 1 to 2 as mild pain, 3 to 6 as moderate pain, and ≥ 7 as severe pain.^[20]^ The assessment of dizziness was represented as yes or no.

Besides, the Riker sedation agitation scale (SAS) was performed to evaluate the status of patients after regaining consciousness:^[21]^ 1 score: patients were unable to be awakened, with no or less response to noxious stimulation, and unable to communicate or obey commands; 2 scores: patients were very quiet with a recovery of physical movement, hardly to be awakened, or to be awakened only responding to physical stimulation, and unable to communicate or obey commands; 3 scores: patients were quiet and can be awakened responding to verbal stimulation or physical shaking with an ability to obey simple commands, but were asleep when stimulation was stopped; 4 scores: patients were calm and cooperative, easily to be awakened with an ability to obey commands; 5 scores: patients were anxious with mild agitation and an ability to obey commands; 6 scores: patients were very restless and unable to be calm down responding to repeated reminders and persuasion, and need to be tied up to prevent them biting endotracheal tube; 7 scores: patients were restless and dangerous, attempting to pluck off endotracheal tube or catheter, even climbing over the bed rails.

### 2.8 Follow-up

Telephone follow-up was conducted for all patients and their families. Outpatient follow-up was performed for survivals. To improve the follow-up rate and get maximum real information of patients, routine outpatient follow-up and 4 fixed follow-up in the first week of 3rd, 6th, 9th, 12nd months were implemented. The follow-up of all patients should be completed within a week. Outpatient follow-up was performed if patients came to the hospital, and telephone follow-up would be applied if they could not come to hospital. All the required information should be collected by telephone and recorded continuously for 3 years until January 2015. The 3-year relapse rate and 3-year survival rate were analyzed.^[22]^

### 2.9 Statistical analysis

All data analyses were performed with Statistical Package for the Social Sciences (SPSS) software (SPSS Inc, Chicago, IL). Measurement data were represented as mean ± standard deviation (mean ± SD). Differences between two groups were tested by one-way analysis of variance (ANOVA). Welch method was used if there was heterogeneity of variance. Dunnetts T3 test was applied for post-hoc multiple comparisons among groups. The *χ*^2^ test was applied to detect the enumeration data. Kaplan–Meier analysis was used to estimate the survival rates of patients in both GA and CEGA groups and log-rank test was applied to compare the survival rate of patients between 2 groups. *P* < 0.05 was regarded as statistically significant.

## 3 Results

### 3.1 Comparisons of clinicopathological features and intraoperative baseline features of GBC patients between the CEGA group and the GA group

There was no significant difference concerning age, weight, gender, ASA classification and TNM stage, period of anesthesia induction, period of anesthesia maintenance, duration of operation, intraoperative urinary output, and blood loss of patients between 2 groups (all *P* > 0.05) (see Table 1).

**Table 1:**
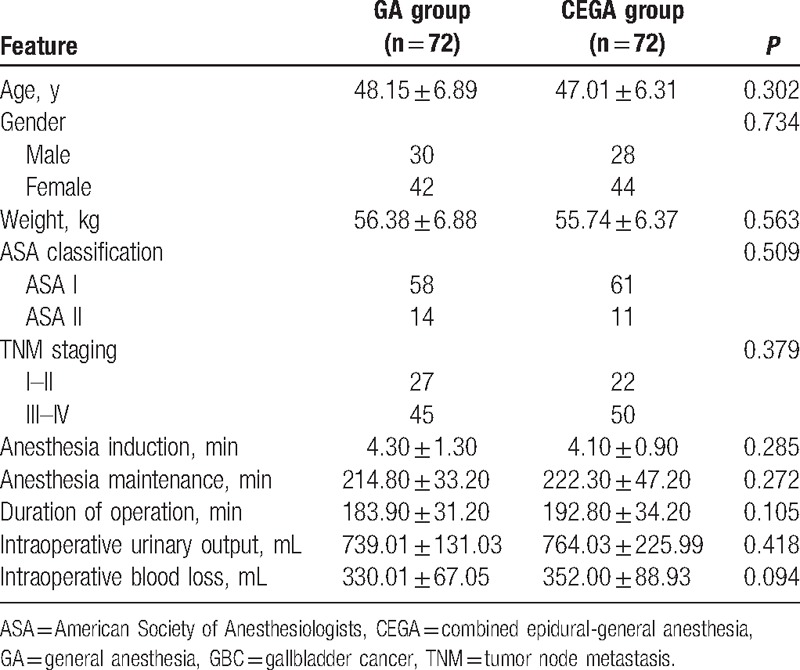
Comparisons of clinicopathological features and intraoperative baseline features of GBC patients between the GA group and the CEGA group.

### 3.2 Comparisons of intraoperative hemodynamic parameters of GBC patients between the CEGA group and the GA group

As shown in Table 2, SBP values at different time points (t1, t2, t3, t4, t5, t6, and t7) were lower than that at the time point of t0 in the GA group (*P* < 0.05). In the CEGA group, the SBP value at t7 was lower than that at t0 (*P* > 0.05), and SBP values at other 6 time points (t1, t2, t3, t4, t5, and t6) were all lower than that at the time point of t0 (all *P* < 0.05). DBP values of the GA group at the time points of t1, t5, and t6 were lower than that at the time point of t0 (*P* < 0.05). The DBP value of the CEGA group at t0 was higher than that at time points of t1, t3, t4, t5, t6, and t7 (all *P* < 0.05). Compared with the time point of t0 in the GA group, decreased HR values were observed at the time point of t1, t3, t5, and t7 (all *P* < 0.05). In CEGA group, HR values at the time point of t1, t3, t4, and t5 were markedly lower than that at the time point of t0 (all *P* < 0.05). BIS values of both the GA and CEGA groups at different time points (t1, t2, t3, t4, t5, t6, and t7) were lower than those at the time point of t0 (all *P* < 0.05).

**Table 2:**
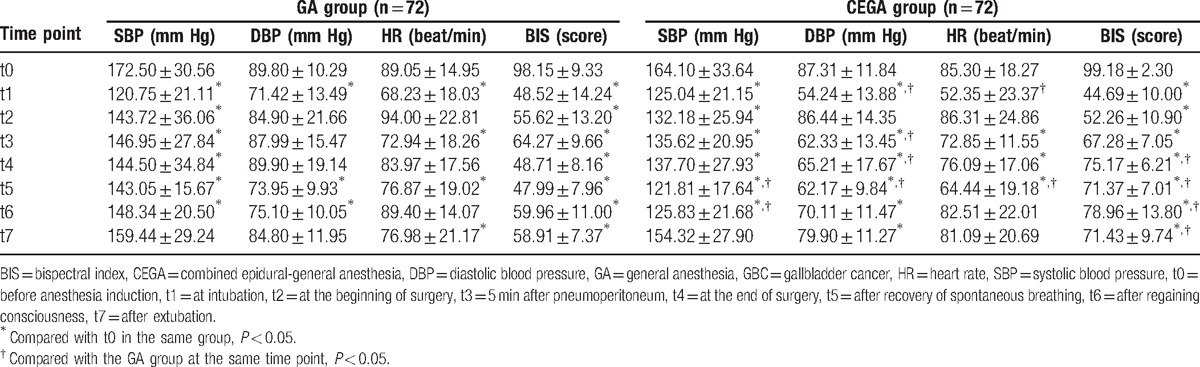
Comparisons of intraoperative hemodynamic parameters of GBC patients between the CEGA group and the GA group.

Comparisons of these values between 2 groups at the each time point are also shown in Table 2. The results indicated that SBP values of the CEGA group at the time points of t5 and t6 were lower than the GA group (*P* < 0.05). DBP values of the CEGA group at the time points of t1, t3, t4, and t5 were lower than the GA group (*P* < 0.05). HR values of the CEGA group at the time points of t1 and t5 were significantly lower than the GA group (*P* < 0.05). BIS values of the CEGA group at the time points of t4, t5, t6, and t7 were significantly higher than the GA group (*P* < 0.05).

### 3.3 Comparisons of postoperative adverse reactions of GBC patients between the CEGA group and the GA group

Comparisons of postoperative adverse reactions among patients between 2 groups are shown in Table 3. Two patients with vomiting and 3 patients with bucking response were observed in the GA group. No vomiting and bucking was observed in the patients of the CEGA group. In the GA group, there were 2 patients with chest tightness and 2 patients with dizziness for patients. No incidence of these adverse reactions was observed in patients of the CEGA group. There was no significant difference of the incidence rates of vomiting, chest tightness, bucking, dizziness, and SAS values between 2 groups (all *P* > 0.05). Compared with the GA group, the mean VAS value of patients in the CEGA group was significantly alleviated (*P* < 0.05).

**Table 3:**
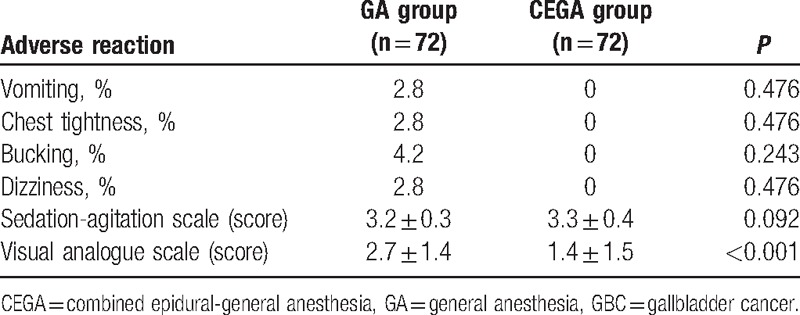
Comparisons of postoperative adverse reactions of GBC patients between the GA group and the CEGA group.

### 3.4 Comparisons of T cell subsets and NK cell survival rates of GBC patients between the CEGA group and the GA group

The survival rates of T cell subsets (CD3+, CD4+, and CD4+/CD8+) exhibited a decreasing tendency in the GA groups at the time points of T3, T4, and T5 relative to these at the time point of T2. And survival rates of CD4+ and CD4+/CD8+ cells exhibited a decreasing tendency in the CEGA group at the time points of T3, T4, and T5 relative to these at the time point of T2. In the GA group, compared with the survival rates of CD3+, CD4+, and CD4+/CD8+ cells at the time point of T1, these at the time points of T3–T5 were significantly reduced (all *P* < 0.05). In the CEGA group, the survival rate of CD3+ at the time point of T3 was significantly lower than that at the time point of T1 (*P* < 0.05), and survival rates of CD4+ and CD4+/CD8+ cells at the time points of T2, T3, T4, and T5 were significantly lower than these at the time point of T1 (all *P* < 0.05).

**Figure 1:**
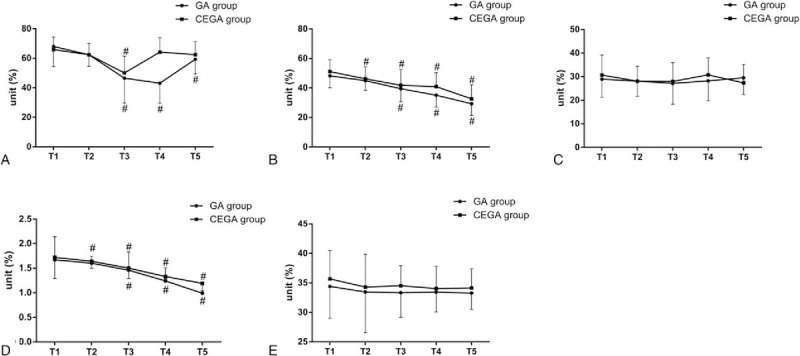
The survival rates of T cell subsets and NK cells at different time points among patients of the GA and CEGA groups. A, The survival rates of CD3+ cells at time points of T1, T2, T3, T4, and T5 among patients of the GA and CEGA groups. B, The survival rates of CD4+ cells at time points of T1, T2, T3, T4, and T5 among patients of the GA and CEGA groups. C, The survival rates of CD8+ cells at time points of T1, T2, T3, T4, and T5 among patients of the GA and CEGA groups. D, The survival rates of CD4+/ CD8+ cells at time points of T1, T2, T3, T4, and T5 among patients of the GA and CEGA groups. E, The survival rates of NK cells at time points of T1, T2, T3, T4, and T5 among patients of the GA and CEGA groups. CEGA = combined epidural-general anesthesia, GA = general anesthesia, NK = natural killer, T1 = 0 min before anesthesia induction, T2 = 2 h after the beginning of surgery, T3 = at the end of surgery, T4 = 1 day after surgery, T5 = 3 days after surgery; ∗, compared with the GA group at the same time point, *P* < 0.05; #, compared with T1 in the same group, *P* < 0.05.

At the time points of T2, T3, T4, and T5, the survival rates of CD3+, CD4+, and CD4+/CD8+ cells of the GA group were lower than those in the CEGA group. Importantly, there were significant differences of the survival rates of CD3+ and CD4+ cells at the time point of T4 between the GA group and the CEGA group (both *P* < 0.05). And the CEGA group had an evidently elevated survival rate of CD4+/CD8+ cell at the time point of T5 than the GA group at the same time point (*P* < 0.05). At the same time points (T1, T2, T3, T4, or T5), there was no distinctive difference of CD8+ cell survival rate between the 2 groups (*P* > 0.05). The survival rates of NK cells between the 2 groups at the same time points (T1, T2, T3, T4, or T5) showed no significant difference (all *P* > 0.05). In the same group (the GA or CEGA group), the survival rates of NK cells at different time points (T2, T3, T4 and T5) were similar to these at the time point of T1 (all *P* > 0.05). Detailed information is shown in Fig. 1 and Table 4.

**Table 4:**
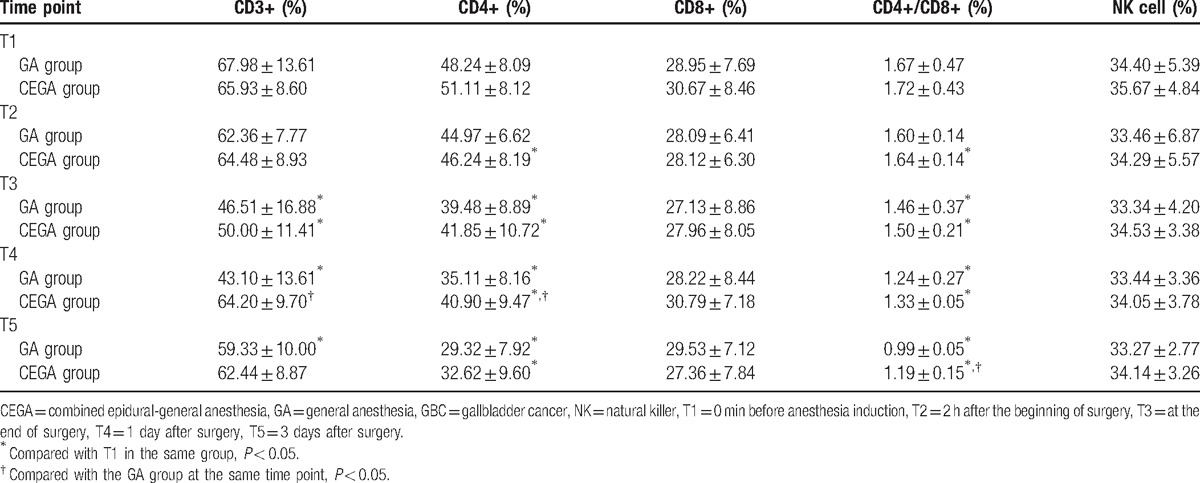
Comparisons of T cell subsets and NK cell survival rates of GBC patients between the GA group and the CEGA group.

### 3.5 Comparison of 1-year, 2-year, and 3-year survival rates of GBC patients between the CEGA group and the GA group

**Figure 2:**
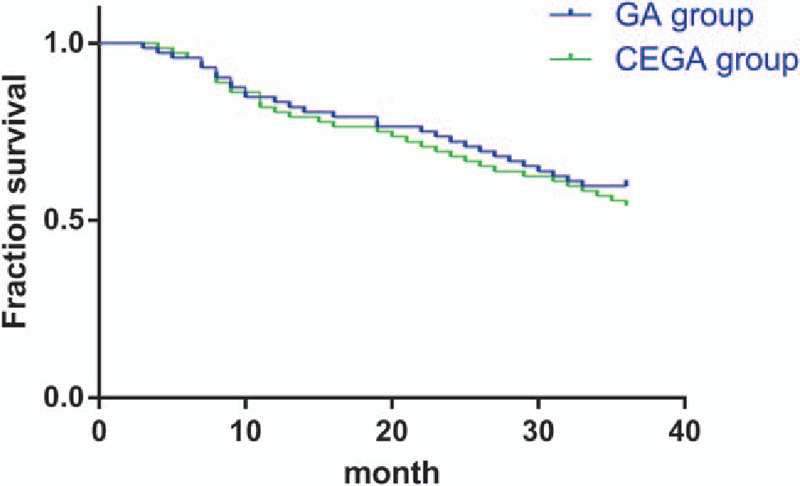
The Kaplan–Meier survival curve for the 3-year survival rates of patients in the GA and CEGA groups. CEGA = combined epidural-general anesthesia, GA = general anesthesia.

No patient was died during the hospitalization. Six patients were died within 6 months after surgery. Both the GA and CEGA groups had a same 6-month survival rate (95.83%; 3/72). The 1-year survival rate of the GA group was 83.33% (12/72) and that of the CEGA group was 80.56% (14/72). The overall 1-year survival rate was 81.94% (26/144). The 2-year survival rate of the GA group was 72.22% (20/72) and that of the CEGA group was 68.06% (23/72). The overall 2-year survival rate was 70.14% (43/144). The 3-year survival rate of the GA group was 59.72% (29/72) and that of the CEGA group was 54.17% (33/72). The overall 3-year survival rate was 56.94% (62/144). The Kaplan–Meier survival curve of the 2 groups is shown in Fig. 2. Log-rank test was performed (*P* = 0.541), and there was no significant difference concerning the 3-year survival rate between the GA group and the CEGA group.

## 4 Discussion

Different anesthesia methods had different effects on the function of immune cells.^[23]^ An anesthesia method with minimum effect on cellular immune function could release patient's pain to its maximum extent.^[14]^ In comparison to general anesthesia, this study explored the effects of combined epidural-general anesthesia on intraoperative hemodynamic responses, postoperative cellular immunity, and prognosis.

This study showed that the survival rates of CD3+, CD4+, CD4+/CD8+ cells of patients in the CEGA group at the time points of T2–T5 were significantly higher than those in the GA group at the same time point, which indicated that the combined epidural-general anesthesia improved cellular immunity after laparoscopic cholecystectomy relative to the general anesthesia. This study measured survival rates of T cell subsets (CD3+, CD4+, CD4+/CD8+) and NK cells before, during, and after the surgery, and found that CD3+, CD4+, CD4+/CD8+ cells of patients with combined epidural-general anesthesia were increased in contrast to these of patients with general anesthesia. The immune function of the body was consistent with the cell level of CD3+. Therefore, the elevated CD3+ level could increase the corresponding immune function of the body.^[24]^ CD4+ cell, which could secret a large number of cytokines and assist CD8+ to kill tumor cells, plays a crucial role in the antitumor immunity.^[25]^ When the cellular immune function was recovered or improved after the surgery, CD4+ was increased first, followed by an increase of CD4+/CD8+ cells. When the cellular immune function was severely damaged after the surgery, the numbers of CD4+ and CD8+ cells changed along with balance destruction between 2 groups. The cell immune disorders and dysfunction occurred with the reduction of immune cells.^[26]^ The increased volume of NK cells could improve immune function activity of the body, which contributes to a capacity of killing tumor cells.^[27,28]^ Several anesthetics lead to an inhibition of cellular immunity, such as sufentanil, remifentanil, and dexmedetomidine, which indicates a correlation of cellular immunity with anesthesia.^[29,30]^ Thus, an appropriate anesthesia method is vital for patients with cholecystectomy. In this study, both 2 anesthesia methods had suppression on the cellular immune function of patients with laparoscopic cholecystectomy. But the results showed less inhibition of T cell immunity of patients with combined epidural-general anesthesia. Thus, the combined epidural-general anesthesia might be a better choice for GBC patients.

Another important result in the study revealed that SBP, DBP, and HR values were prominently lower in the CEGA group than that in the GA group. Additionally, our findings indicated lower incidences of adverse reactions and VAS score in the CEGA group in comparison to the GA group, implying that combined epidural-general anesthesia is more effective and safe during the cholecystectomy. To our best knowledge, higher SBP is considered an important risk factor in the cancer.^[31]^ And higher DBP is also associated with the cognitive impairment.^[32]^ In consistent with our results, Khajavi et al^[33]^ conducted a research with regard to combined epidural-general anesthesia versus general anesthesia in elective lumbar spine disk surgery, and they demonstrated that the combined anesthesia contributed to lower mean arterial blood pressure and HR, as well as reduced pain score in the combined epidural-general anesthesia group during the postoperative period. Interestingly, Senoglu et al^[34]^ demonstrate that the patients exhibit remarkably lower heart-rate and blood-pressure levels with combined epidural analgesia and general anesthesia during laparoscopic cholecystectomy than these with the general anesthesia. As Pei et al^[35]^ have reported that combined epidural-general anesthesia results in a better clinical outcome through reducing the dosage of anesthetic agents to promote the intact and sustained immunological function. Based on these findings, this study further proved that combined epidural-general anesthesia contributed to more stable hemodynamic responses during surgery, and attenuated adverse reactions after surgery. Although the present study found that the survival rate of patients in the CEGA group is slightly higher than that in the GA group, this result is limited with small sample size and short-term follow-up, which still needs to be confirmed in future studies.

In summary, the combined epidural-general anesthesia might attenuate intraoperative hemodynamic responses and adverse reactions, and improve postoperative cellular immunity. The combined epidural-general anesthesia was more suitable for the immune function protection of GBC patients with a more satisfactory therapeutic effect, thereby improving the living quality and life quality of patients. However, the sample size of this study is quite small, and the follow-up is not so long to provide a stronger evidence for this finding. Furthermore, this study did not mention the immune function of the patients with noncancer cholecystectomy. Thus, more evidences should be provided with larger sample and longer follow-up in future, and a comparison of immune function between noncancer cholecystectomy and cancerous ones should also be performed.

## Acknowledgments

The authors show their gratitude for helpful comments on this paper received from their reviewers.
